# Characterization of an *Escherichia coli* Isolate Coharboring the Virulence Gene *astA* and Tigecycline Resistance Gene *tet*(X4) from a Dead Piglet

**DOI:** 10.3390/pathogens12070903

**Published:** 2023-07-03

**Authors:** Jianmei Wang, Yuting Huang, Chunjiu Guan, Jie Li, Hua Yang, Guoping Zhao, Canying Liu, Jiangang Ma, Biao Tang

**Affiliations:** 1 State Key Laboratory for Managing Biotic and Chemical Threats to the Quality and Safety of Agro-Products & Institute of Agro-Product Safety and Nutrition, Zhejiang Academy of Agricultural Sciences, Hangzhou 310021, China; wangjianmei@zaas.ac.cn (J.W.); huangyt0303@163.com (Y.H.); guanchunjiu@163.com (C.G.); yanghua@zaas.ac.cn (H.Y.); 2 School of Life Science and Engineering, Foshan University, Foshan 528225, China; liucy3032@163.com; 3 College of Life Science, Liaocheng University, Liaocheng 252000, China; lijie@lcu.edu.cn; 4 School of Life Science, Hangzhou Institute for Advanced Study, University of Chinese Academy of Sciences, Hangzhou 310024, China; gpzhao@sibs.ac.cn

**Keywords:** *Escherichia coli*, *tet*(X4), *astA*, complete genome sequence

## Abstract

*tet*(X4) is the critical resistance gene for tigecycline degradation that has been continually reported in recent years. In particular, pathogenic bacteria carrying *tet*(X4) are a severe threat to human health. However, information describing *Escherichia coli* coharboring *tet*(X4) with virulence genes is limited. Here, we isolated an *E. coli* strain coharboring *tet*(X4) and the heat-stable toxin gene *astA* from a dead piglet. The strain named 812A1-131 belongs to ST10. The genome was sequenced using the Nanopore and Illumina platforms. The virulence genes *astA* and *tet*(X4) are located on the chromosome and in the IncHI1-type plasmid p812A1-tetX4-193K, respectively. The plasmid could be conjugatively transferred to recipient *E. coli* J53 with high frequency. In vivo experiments showed that strain 812A1-131 is pathogenic to *Galleria mellonella* and could colonize the intestines of mice. In summary, pathogenic *E. coli* could receive a plasmid harboring the *tet*(X4) gene, which can increase the difficulty of treatment. The prevalence and transmission mechanisms of pathogenic bacteria coharboring the *tet*(X4) gene need more attention.

## 1. Introduction

Antimicrobial resistance (AMR) has become a global public health problem. The emergence of multidrug-resistant (MDR) bacteria is threatening human health. Tigecycline is a third-generation tetracycline known as the last resort to treat MDR bacterial infections in hospitals [1]. Unfortunately, the tigecycline resistance gene *tet*(X) has emerged and spread widely in animal-derived bacteria, which may be accelerated by the overuse and misuse of tetracycline antibiotics in livestock and poultry [2]. Tigecycline-resistant bacteria harboring the *tet*(X4) gene have also been discovered in patients, limiting the antibiotic treatment and threatening human health [3]. 

The *tet*(X) gene family encoding flavin-dependent monooxygenase can degrade all tetracycline antibiotics, including tigecycline, omadacycline, and eravacycline. In recent years, plasmid-mediated *tet*(X4) gene transfer among animal-derived bacteria has caused wide public concern [4]. Notably, the IncX1, IncX3, IncHI1, and IncQ plasmids carrying the *tet*(X4) gene are most common and have been identified in Enterobacteriaceae [5,6,7]. *Escherichia coli* harboring *tet*(X4) have been isolated from multiple sources, including birds, retail meat, and the environment, and they have also been found in human clinical samples [8,9,10,11,12].

*E. coli* is a common opportunistic zoonotic pathogen. *E. coli* carrying virulence genes can cause human and animal diseases, such as gastroenteritis, cholecystitis, urinary tract infections (UTIs), and pneumonia in humans [13,14]. It can also cause severe diarrhea in pigs, chickens, cattle, and other animals, or even death, especially in newborn animals [15,16]. Diarrheagenic *E. coli* (DEC) can be classified into six groups according to their virulence genes: enteropathogenic *E. coli* (EPEC), enterohaemorrhagic *E. coli* (EHEC), shiga-toxin-producing *E. coli* (STEC), enterotoxigenic *E. coli* (ETEC), enteroaggregative *E. coli* (EAEC), enteroinvasive *E. coli* (EIEC), and diffusely adherent *E. coli* (DAEC) [17]. The *eae*, *stx*, *elt*, *estB*, *faeG*, *aggR*, *ipaH*, and *Afa*-Dr adhesin genes are the main virulence genes in these pathogens [18,19]. The *astA* virulence gene encodes a heat-stable toxin that is widespread in pathogens, such as EAEC, ETEC, STEC, and EHEC [20]. *E. coli* carrying the *astA* virulence gene are associated with diarrhea and the enhanced pathogenicity of other virulence factors. Such strains can cause severe gastrointestinal disease in animals and lead to economic losses [21].

Multilocus sequence typing (MLST) is an important method for analyzing specific bacterial typing based on different housekeeping genes. MLST analysis could be helpful in understanding the processes of genomic evolution in diverse species and the characteristics of a specific clone [22]. *E. coli* ST types are extraordinarily diverse, including ST10, ST48, ST95, and so on [23]. *E. coli* ST10, as a non-host-restricted pathogenic bacterium, can spread in humans and animals [24]. ST10 strains are common among MDR *E. coli* and have always carried various virulence genes, including *gad* (glutamate decarboxylase), *iss* (increased serum survival), *terC* (tellurium ion resistance protein gene), and *sitA* (iron transport protein) [25,26]. It should be noted that *mcr-1*, *tet*(X4), and *bla*_NDM-5_ have been detected in ST10, which showed that ST10 strains are important carriers for ARGs [27]. Moreover, some ST10 strains belonging to the DEC have been discovered in diarrheal pigs and patients [4,28]. DEC ST10 carrying ARGs in sick animals and patients would increase the risk of antibiotic treatment failure.

In a routine antimicrobial resistance surveillance, we isolated an *E. coli* strain cocarrying the resistance gene *tet*(X4) and the virulence gene *astA* from a piglet of unknown cause of death. We tested the AMR of the strain and analyzed the genome characterization after whole-genome sequencing to elucidate the transmission mechanism of the *tet*(X4) gene and the virulence. The virulence of strain 812A1-131 was detected by in vivo experiments in *Galleria mellonella* and mice. This strain may pose a threat to other animals and humans. 

## 2. Materials and Methods

### 2.1. Pathogen Detection

An anal swab of a dead pig (of unknown cause of death) at 18 days of age was sent from a farm. The RNA in the feces was extracted according to the instructions for the Column Stool RNAOUT kit (Yaji biological Co., Ltd., Shanghai, China). The RNA was immediately subjected to reverse-transcription PCR (Vazyme Biotech Co., Ltd., Nanjing, China) using the primer Oligo dT. Then, the RT-PCR amplified procedure and the detection primers for porcine epidemic diarrhea virus (PEDV), porcine transmissible gastroenteritis virus (TGEV), and porcine rotavirus (RV) were used as described in a previous study [29].

### 2.2. Bacterial Isolation and Identification

The swab was put into a sterile tube containing 5 mL BPW (Hangzhou Microbial Reagent Co., Ltd., Hangzhou, China) to enrich the bacteria and incubated at 37 °C at 200 rpm for 12–18 h. The bacterial solution was scribed onto MacConkey agar with a disposable inoculating loop and statically incubated at 37 °C for 12–18 h. A single colony on the McConkey agar plate was inoculated on eosin methylene blue (EMB) agar and Luria–Bertani (LB) medium and incubated overnight at 37 °C. The culture mediums were purchased from Beijing Land Bridge Technology Co. Ltd., Beijing, CHN. The strain was identified by PCR using the primers described in a previous study [30]. The stain was named 812A1-131 and preserved in a cryovial containing 1 mL of 25% glycerol at −80 °C.

### 2.3. Antimicrobial Susceptibility Testing

The broth dilution method was used to determine the minimum inhibitory concentration (MIC) of strain 812A1-131 according to the Clinical and Laboratory Standards Institute (CLSI) recommendations. *E. coli* ATCC25922 was used as a quality control, as previously described [31]. A total of 13 antibiotics were selected for antimicrobial susceptibility testing, including ampicillin (AMP), amoxicillin/clavulanic acid (A/C), cefotaxime (CTX), meropenem (MEM), amikacin (AMK), gentamicin (GEM), colistin (COL), ceftiofur (CEF), ciprofloxacin (CIP), trimethoprim/sulfamethoxazole (SXT), tetracycline (TET), tigecycline (TIG), and florfenicol (FFC). The antibiotics were purchased from Biofosun, Fosun Diagnostics, Shanghai, China.

In short, the concentrated antibiotic solutions were diluted, and 100 μL volumes were added to 96-well plates by double dilution according to the CLSI instructions. Then, 2–5 single colonies of *E. coli* were scraped from a fresh LB agar plate and adjusted to 0.5 McFarland standard inoculum (1.5 × 10^8^ CFU/mL) using saline solution. The suspension was added to MH nutrient broth at a ratio of 1:200, and then 100 μL of diluent was added to each well. The 96-well plate containing the antibiotics and bacterial suspension was placed in an incubator at a constant temperature of 37 °C for 16–18 h.

The MIC values of tigecycline were verified by the agar dilution method and E-test assay (Tang et al., 2022). *E. coli* ATCC 25922 was used as a quality control. Briefly, serial dilutions of bacterial suspensions (10^−1^~10^−6^) were inoculated onto LB agar plates with different concentrations of tigecycline and subsequently incubated at 37 °C for 12 h [32]. 

### 2.4. Whole-Genome Sequencing (WGS) and Bioinformatics Analysis

Whole-genome sequencing is a valuable way to analyze ARGs and virulence factors and has been used in the diagnosis of animal pathogens [33,34,35,36]. The genome of the tigecycline-resistant strain was extracted using the Generay DNA kit (Generay, Shanghai, China). WGS was performed using the Illumina HiSeq and Nanopore sequencing platforms. Unicycler v.0.4.3 was used for assembly, and RAST (https://rast.nmpdr.org/ (accessed on 17 January 2023)) was used for gene annotation [37]. The Center for Genomic Epidemiology (https://cge.cbs.dtu.dk//services/ (accessed on 17 January 2023)) was used to analyze ARGs with ResFinder 4.1 [38]. PlasmidFinder 2.1 and VirulenceFinder 2.0 were used to predict plasmid replicon and virulence genes [39]. The serotype was predicted by SerotypeFinder 2.0 (https://cge.cbs.dtu.dk/services/SerotypeFinder/ (accessed on 17 January 2023)) [40]. BacWGSTdb was used to observe the geographical distribution of closely related plasmids and, based on the cgMLST strategy to generate a phylogenetic tree, analyze the relationship between resistance genes of similar strains [41]. The plasmids were visualized using BRIG [42].

### 2.5. Conjugative Transfer

The sodium-azide-resistant *E. coli* strain J53 was used as the recipient, and the *tet*(X4)-positive strain 812A1-131 was used as the donor. *E. coli* J53 is sensitive to tigecycline, as demonstrated in previous studies [5]. Strain 812A1-131 was sensitive to sodium azide and could not grow in LB agar plates containing sodium azide (100 mg/L). The bacteria were cocultured at two temperatures, 37 °C or 28 °C, for 16 h. The mixed culture was diluted to 10^−1^~10^−6^ using PBS, and 10 μL of the above diluted solution was inoculated on LB agar plates containing tigecycline and sodium azide. The LB agar plates were incubated at 37 °C for 12~16 h [31]. Single colonies of transconjugants were randomly selected, and the *tet*(X4) gene was verified by PCR [5].

### 2.6. S1-PFGE and Southern Blotting

The *tet*(X4) strain was embedded and subsequently lysed using SeaKem Gold Agarose (Lonza Rockland, Inc., Maryland, MD, USA), with *Salmonella* H9812 as the marker, as described previously [32]. The treated DNA fragments were then separated at 14 °C for 18 h in 0.5× Tris-borate EDTA buffer. A pulse-field electrophoresis system (CHEF Mapper, Bio-Rad Laboratories, California, CA, USA) was used with a voltage of 6 V, an electric field angle of 120°, and a pulse time of 2.2 to 63.8 s. After the gel was removed at the end of the procedure, the gel blocks were stained and observed with a gel imager. The *tet*(X4)-specific probe was labeled according to the instructions of the DIG High Prime DNA marker and assay starter kit (Roche Diagnostics GmbH, Mannheim, Germany).

### 2.7. The Galleria mellonella Model and the Mouse Infection Model

To evaluate the virulence of 812A1-131, the *Galleria mellonella* infection model was used to conduct in vivo experiments. *E. coli* 649A1 containing the *stx2* gene was used as the virulent control and stored in the laboratory. Sterilized PBS served as a blank control. The inactivated 812A1-131 strain (incubated at 65 °C for 30 min) and the avirulent strain DH5α were used as the negative controls to assess the virulence of the live strain 812A1-131 [43]. Larvae with symmetrical physiques were evenly divided into the PBS control group, the experimental group, the highly virulent control group, and the avirulent control group. Ten larvae were set in each group, and the larvae were injected with 10 μL PBS or 10^6^ CFU/mL bacterial inoculum, as previously described [44]. The death of larvae in each group was recorded every 6 h and observed for 48 h. GraphPad software was used for the plotting and statistical analysis of the data. 

The mouse infection model test was performed as described in a previous report [45]. Ten 5-week-old SPF ICR male mice (Hangzhou Qizhen Laboratory Animal Technology Co., Ltd., Hangzhou, China) were weighed (about 25 g per mouse) and randomly divided into two groups, with five animals in each group. The bacterial solution (10^9^ CFU in 100 μL) or PBS containing 20% sucrose were injected into the stomachs of the mice. To obtain the highest number of *E. coli*, the mice were weighed and euthanized by cervical dislocation after 6 h, as previously described [46]. The ileum, cecum, and colon parts were collected. Moreover, the tissue was vigorously cleaned and ground with PBS. The homogenate was diluted 1000 times using PBS, and 100 μL was coated on a MacConkey plate (containing 2 μg/mL tigecycline) at 37 °C for 16 h. Then, colony counting was performed.

## 3. Results

### 3.1. Pathogen Detection and Bacterial Isolation

PEDV, TGEV, and RV were not detected in the dead pig. The cause of the pig’s death could not be determined. An E. coli strain was successfully isolated and identified by PCR.

### 3.2. Antimicrobial Susceptibility

**Figure 1:**
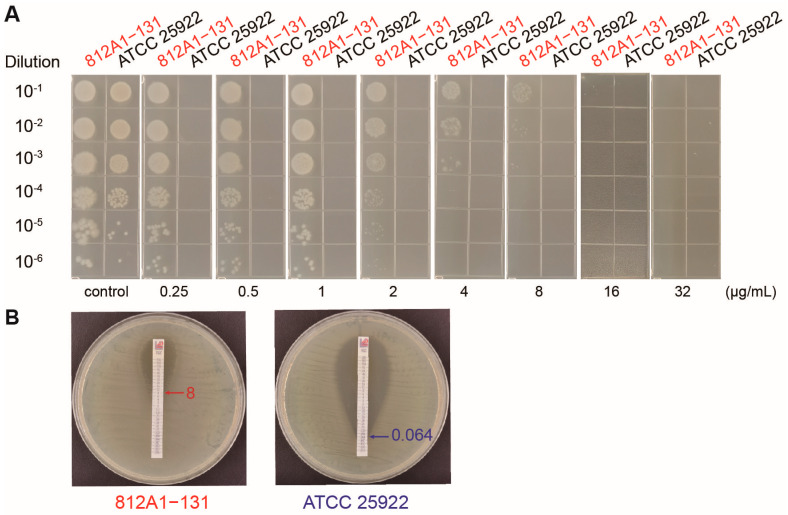
The antimicrobial resistance of strain 812A1-131 to tigecycline. (**A**) The growth of strain 812A1-131 on LB plates with different tigecycline concentrations. (**B**) E-test analysis of strain 812A1-131. *E. coli* ATCC 25922 was used as the control.

Antimicrobial susceptibility testing showed that strain 812A1-131 was only sensitive to meropenem and trimethoprim/sulfamethoxazole and showed an intermediate reaction to colistin (Table 1). It was resistant to tigecycline, with an MIC of 16 μg/mL. The agar dilution method and E-test were used to further verify its resistance to tigecycline (Figure 1). Strain 812A1-131 is a multidrug-resistant (MDR) strain that is resistant to 10 different antibiotics from nine classes (AMP-A/C-CTX-AMK-GEM-CEF-CIP-TET-TIG-FFC).

### 3.3. Whole-Genome Sequencing and Sequence Analysis

**Figure 2:**
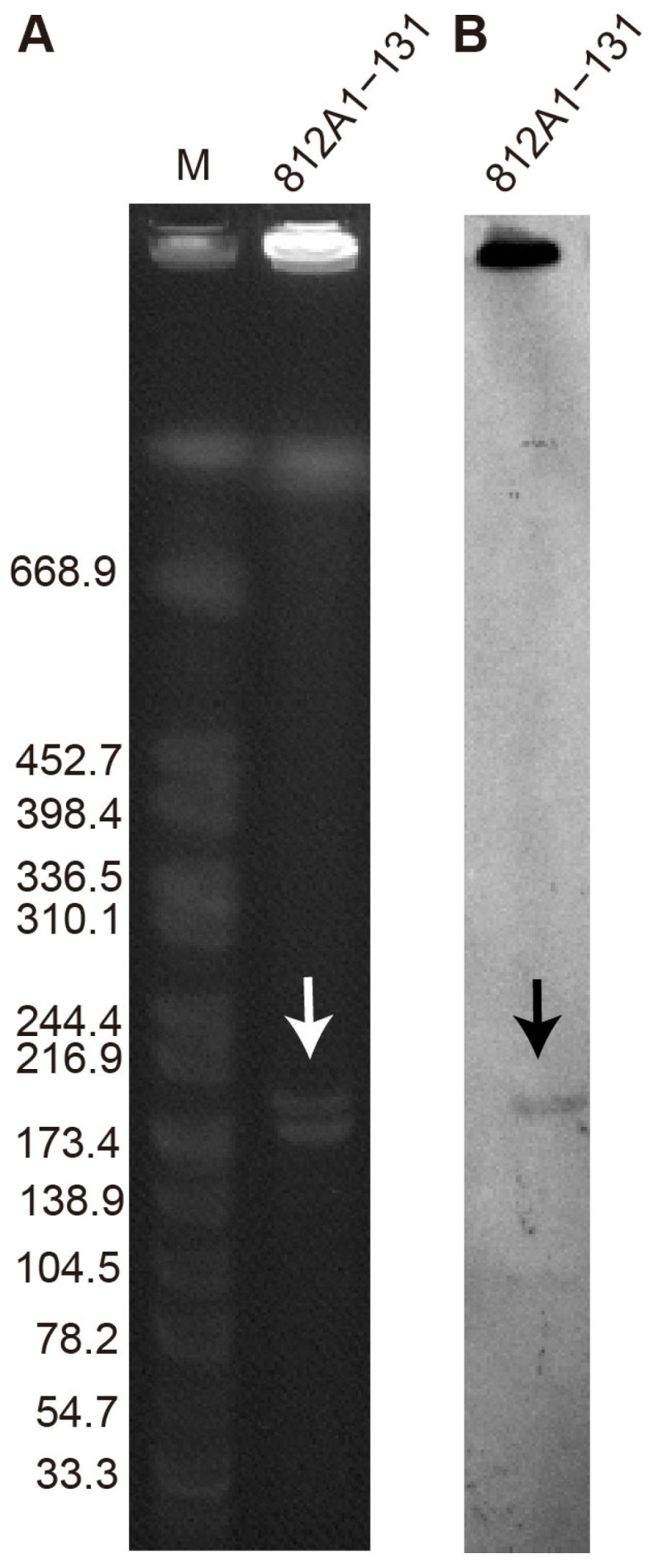
S1-PFGE (**A**) and Southern blot (**B**) of strain 812A1-131. The plasmid harboring the *tet*(X4) gene is indicated with arrows.

The strain 812A1-131 has a genome consisting of one chromosome and three plasmids. The chromosome size was 4,665,801 bp, and the GC content was 50.9%. The MLST analysis showed that the strains belonged to *E. coli* ST10. There are three plasmids located in 812A1-131 that were named p812A1-tetX4-193K, p812A1-69K, and p812A1-65K. The plasmid p812A1-tetX4-193K has a size of 193,145 bp and a GC content of 46.21%, and it contains two types of replicons (IncHI1A and IncHI1B(R27)). p812A1-69K has no known replicon and is 69,262 bp with a 51.89% GC content. The size of p812A1-65K is 65,072 bp, and it contains the IncFIA(HI1)- and IncY-type replicons. In addition, the size of plasmid p812A1-tetX4-193K was verified using S1-PFGE, which was consistent with the whole-genome sequencing analysis (Figure 2).

**Figure 3:**
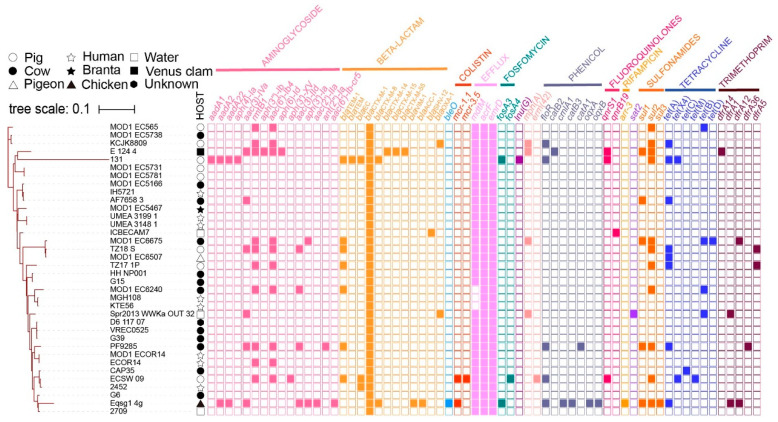
Phylogenetic tree of ST10 strains similar to 812A1-131 based on SNPs of core genomes.

**Figure 4:**
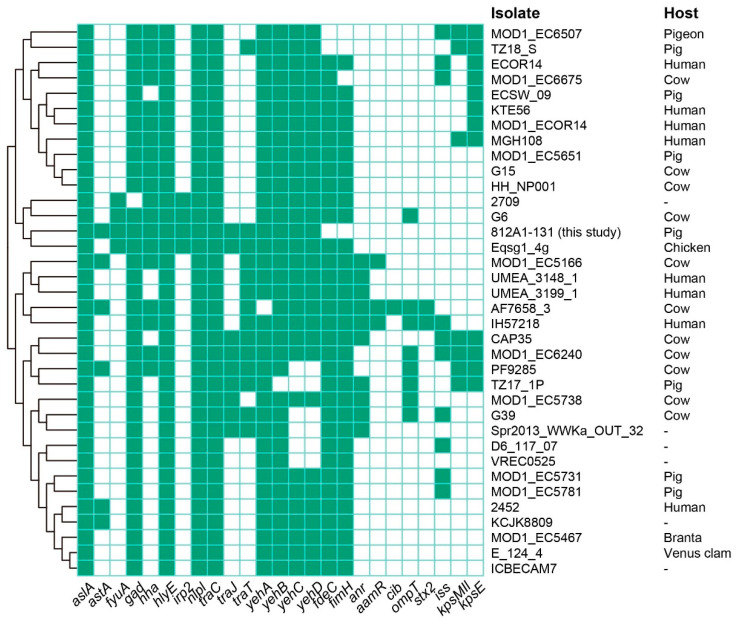
Virulence gene profiles of ST isolates closely related to 812A1-131.

Sixteen antimicrobial resistance genes (ARGs) were identified in 812A1-131, of which six were located on p812A1-tetX4-193K: *tet*(X4), *bla*_TEM-1B_, *lnu*(G), *aadA22*, *qnrB*, *qnrS1*, and *floR*. The plasmid p812A1-65K contains seven ARGs: *fosA3*, *aph(4)-Ia*, *aac(3)-IV*, *sul2*, *tet*(A), *bla*_CTX-M-14_, and *floR*. In addition, *rmtB*, *bla*_TEM-141_, *bla*_CTX-M-55_, and *bla*_TEM-1B_ were located on the plasmid p812A1-69K (Table 1). A total of 16 virulence genes were identified, including *astA*, *hha*, *hlyE*, *traT, yehA*, and *traJ.* These virulence genes are related to heat-stable toxins, hemolysin, outer membrane protein virulence factor, and invasion of the blood–brain barrier, respectively. Strains with close homology (<1000 SNP) from different hosts of ST10 types were screened from the NCBI database. These strains were identified in humans, cows, pigs, chickens, and the environment. The phylogenetic tree showed that strain 812A1-131 is relatively independent compared to the others (Figure 3). Some strains carried multiple ARGs, but the *tet*(X4) gene was rare. Except in strain 812A1-131, the *tet*(X4) gene was also found in strain ECSW_09, which was identified from pigs (Figure 3). Moreover, these strains contained several virulence genes, *alsA*, *csg*, *hlyE*, *nlpl*, and *traC* (Figure 4).

### 3.4. Conjugative Transfer of 812A1-131

The recipient strain *E. coli* J53 was able to grow on an LB agar plate containing sodium azide and tigecycline after coculture with strain 812A1-131, confirming conjugative transfer. The *tet*(X4) gene was located in the transconjugants, which was verified by PCR. This result indicated that, by conjugation, strain 812A1-131 could transfer the plasmid harboring the *tet*(X4) gene to recipient bacteria. The conjugative transfer frequencies were different between different temperatures, being lower at 37 °C (1.87 ± 0.89 × 10^−5^) than at 28 °C (4.71 ± 5.64 × 10^−3^).

### 3.5. Genetic Environment Analysis of p812A1-tetX4-193K

**Figure 5:**
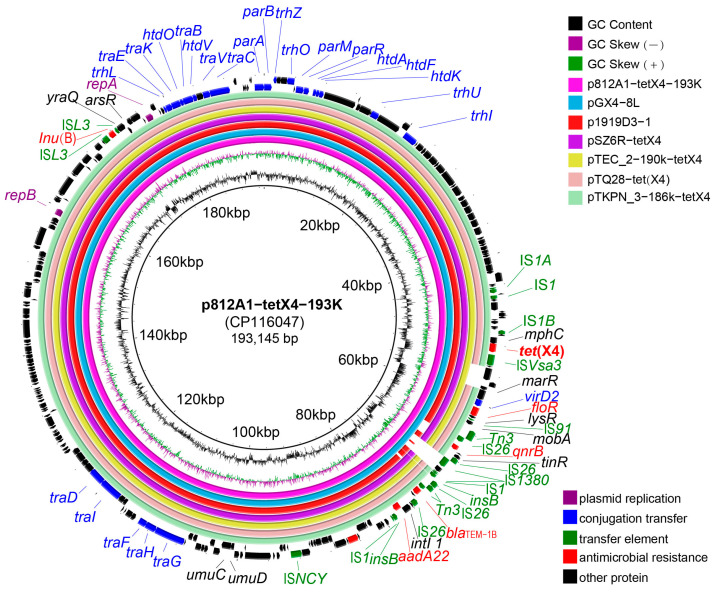
Plasmid characteristics and genetic environment of plasmid p812A1-tetX4-193K. Ring comparison of p812A1-tetX4-193K with similar plasmids was conducted using BRIG.

The complete sequence of plasmid p812A1-tetX4-193K was obtained by sequencing. The highly similar plasmids (coverage > 97%, identity > 99%) compared with p812A1-tetX4-193K from different hosts were screened using BLAST from the GenBank database (Figure 5). They were all IncHI1-type plasmids that were identified from *Enterobacter hormaechei* (pGX4-8L (CP071877)), *Citrobacter sp*. (pSZ6R-tetX4 (MW940627)), *Morganella morganii* (pTQ28-tet(X4) (ON390816)), *Enterobacter cloacae* (pTECL_2-190k-tetX4 (MZ773210)), and *Klebsiella pneumoniae* (pTKPN3-186K-tetX4 (MZ773211)). These strains were isolated from swine feces, swine nasal swabs, and so on. pGX4-8L had the highest similarity to p812A1-tetX4-193K, and the other plasmids were found to lack a segment in the comparisons with p812A1-tetX4-193K (Figure 5). It is worth noting that these five plasmids came from different species in China. The IncHI1 plasmid has a higher risk of spreading the *tet*(X4) gene between bacteria.

### 3.6. Pathogenic Testing of Strain 812A1-131 in Galleria mellonella and Mouse

**Figure 6:**
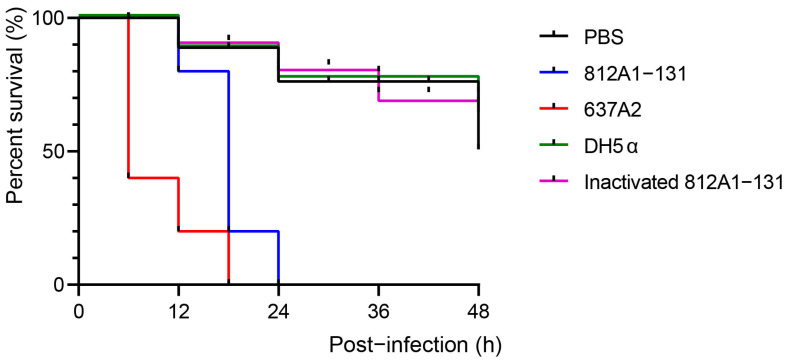
Survival curve of *Galler mellonella.* The black line indicates the *Galler mellonella* survival rate of the PBS control group. The blue and red lines indicate the experimental group (812A1-131) and the highly virulent control group (637A2), respectively. The green (DH5α) and purple (inactivated 812A1-131) lines indicate the two avirulent control groups.

Strain 812A1-131 and the highly virulent *E. coli* strain 629A1 killed 80% and 100% of *Galleria mellonella* at 20 h, respectively. All *Galleria mellonella* died by 24 h after infection with strain 812A1-131. However, only 10% of *Galleria mellonella* died in the PBS or avirulent groups by 20 h. The fatality rate of strain 812A1-131 was significantly higher than that of the PBS and avirulent strains (Figure 6). The mice were subjected to gavage to observe the bacterial colonization ability. The change in weight was not significantly different between the different groups. *E. coli* 812A1-131 was isolated from the ileum, cecum and colon. The numbers of colonies were 2.58 × 10^3^, 2.79 × 10^8^, and 7.11 × 10^6^ CFUs/g, respectively. This strain preferred to colonize the cecum and colon. 

## 4. Discussion

Although tigecycline has not been used in breeding, reports of tigecycline-resistant bacteria carrying *tet*(X4) genes in animals have increased. This may be due to the widespread use of tetracycline in food animals. *E. coli* is an essential host of *tet*(X4) resistance genes. Notice that the host range of *tet*(X4) genes is increasingly extensive, and *tet*(X4) has even been found in pathogenic bacteria [47,48]. The risk of tigecycline therapy failure is increased when an MDR pathogen harboring *tet*(X4) infects humans. In this study, one *E. coli* strain belonging to ST10 carrying the *tet*(X4) resistance gene and the *astA* virulence gene was isolated from a dead piglet.

ST10 is a common *E. coli* sequence type that has been widely isolated from the environment, animals, and even humans [49]. *E. coli* ST10 is a potential food-borne pathogen that threatens human health and has been identified in the food chain [50,51]. There has been no direct evidence of the direct transmission of *E. coli* ST10 to humans through contact or the food chain [52]. However, previous studies have shown that the ST10 *E. coli* strain from the environment has a genome closely related to the strain from humans. The same ST10 was observed in human patients, pig feces, and pork samples [27,53]. This result suggested that ST10 has a broad host range and is a zoonotic pathogen. Moreover, ST10 strains are usually multidrug-resistant (MDR) bacteria [54]. The ST10 strain of *E. coli* has resistance to seven types of antibiotics, including colistin, and was isolated from Polish poultry [55]. MDR ST10 *E. coli* resistance to imipenem and colistin was identified in young clinical patients [56]. 

ARGs can be located on chromosomes or mobile genetic elements (MGEs) of bacteria. MGEs can help to rapidly spread ARGs that include integrons, transposons, insertion sequences, and plasmids [57]. Plasmids were the most common MGEs for transferring ARGs within the same family of bacteria. The *tet*(X4) gene was identified in an IncHI1 plasmid in this study. The IncHI1 plasmid is usually larger than 180 kb, and it always carries various ARGs and heavy-metal resistance genes [58]. It has a wide host range and has been widely isolated from various *Enterobacteriaceae* bacteria, especially *Salmonella* [59]. This plasmid plays an important role in *tet*(X4) transmission and has become the second most-prevalent plasmid for transferring the *tet*(X4) gene. The p812A1-tetX4-193K plasmids from the dead piglet were similar to those from the nonpathogen in different regions. This result indicated that the *tet*(X4)-positive IncHI1 plasmid is widespread and could transfer horizontally between bacteria in humans, animals, food, and the environment [60]. Furthermore, IncHI is a common plasmid type carrying the colistin resistance gene *mcr* [61]. It carries the risk of coharboring the *tet*(X) and *mcr* genes, which would be a serious threat to human health.

The emergence of pathogens with important AMR genes and virulence factors seriously threatens public health. Pathogenic *K. pneumoniae* carrying virulence genes *hvKp* and *tet*(X4) were isolated from pork samples and caused 100% death in mice 12 h after inoculation [62]. Foodborne pathogens can infect humans, threatening human health [63]. The coexistence of the virulence factor *tdh* and the AMR gene *mcr-1* was also discovered in *Vibrio parahemolyticus* [64]. Pathogenic *E.coli* (UPEC) containing the *bla*_NDM_ gene has caused multiple typical urinary tract infections in patients [65]. This result indicated that bacteria coharboring resistance genes and virulence factors are widespread, limiting antibiotic treatment. The *astA* gene has been identified in various DEC strains with outbreaks of diarrhea [66,67,68,69]. This study indicated that the *astA* gene might increase the strain’s virulence. The difficulty of treatment is increased when a pathogen acquires the *tet*(X4) gene. Therefore, monitoring the coexistence of resistance genes with virulence factors in bacteria is very important. Furthermore, limiting the use of tetracycline antibiotics in livestock and poultry may help reduce the spread of resistant bacteria. People should avoid contact with live animals and choose processed meats instead.

In conclusion, an *E. coli* strain coharboring *tet*(X4) and *astA* was isolated from a dead piglet in routine antimicrobial resistance surveillance. The 812A1-131 strain infection could lead to the death of *Galleria mellonella*, which indicates that *astA* might have virulence potential. The IncHI1 plasmid transferred *tet*(X4), which is increasingly common. The acquisition of *tet*(X4) by pathogenic bacteria would increase treatment difficulty in humans and animals, and the bacteria coharboring virulence and AMR genes need attention.

## 5. Accession Numbers

The whole-genome sequence of *E. coli* 812A1-131 has been submitted to GenBank with the accession numbers CP116046-CP116049.

## Figures and Tables

**Table 1:** MIC and AMR genes of strain 812A1-131.

IDs	Antibiotics	812A1-131	
		MIC (μg/mL)	ARGs
1	Ampicillin	>128 ^R^	*bla*_TEM-1B_, *bla*_TEM-141_, *bla*_TEM-206_, *bla*_CTX-M-55_, *bla*_OXA-10_, *bla*_CTX-M-14_
2	Amoxicillin/clavulanic acid	>128/64 ^R^	
3	Cefotaxime	>8 ^R^	*bla*_CTX-M-55_, *bla*_CTX-M-14_
4	Meropenem	1 ^S^	/
5	Amikacin	>64 ^R^	*rmtB*
6	Gentamicin	>32 ^R^	*aac(3)-IV*, *rmtB*
7	Colistin	1 ^S^	/
8	Ceftiofur	>32 ^R^	
9	Ciprofloxacin	>8 ^R^	*qnrS1*, *parC*, *gyrA*
10	Trimethoprim/sulfamethoxazole	>0.5/9.5 ^R^	*sul2*
11	Tetracycline	>64 ^R^	*tet*(X4), *tet*(A)
12	Tigecycline	>16 ^R^	*tet*(X4)
13	Florfenicol	128 ^R^	*floR*

R: resistance; S: susceptibility; /: no AMR gene. The results are based on CLSI guidelines.

## Data Availability

The datasets generated during and/or analyzed during the current study can be find in the main text.
